# Efficacy of Greater Occipital Nerve Blockade in Craniofacial Neuralgia and Facial Pain Syndromes: A Retrospective Chart Review with Prospectively Collected Follow-Up Data

**DOI:** 10.3390/jcm14145034

**Published:** 2025-07-16

**Authors:** Turan Poyraz, Aynur Ozge

**Affiliations:** 1 Department of Elderly Care, Izmir University of Economics, 35330 Izmir, Turkey; 2 Department of Neurology, Headache Clinic, 33430 Mersin, Turkey; aynurozge@gmail.com; 3 NOROM Neuroscience and Excellence Center, Mersin University, 06560 Ankara, Turkey

**Keywords:** headache, cervicogenic headache, greater occipital nerve block, craniofacial neuralgias, facial pain, trigeminal neuralgia, occipital neuralgia, trigeminal neuropathic pain

## Abstract

**Background/Objectives**: Greater occipital nerve blockade (GONB) is a minimally invasive intervention used to treat primary headaches. However, the evidence regarding its role in craniofacial pain syndromes and its potential impact on analgesic use remains limited. Previous studies have reported that GONB is an effective method in patients with cranial neuralgia, but its efficacy is limited in persistent idiopathic facial pain (PIFP). **Methods**: This study was a retrospective cohort trial examining the medical records of 26 patients who applied to our Headache Clinic due to facial pain and cranial neuralgia between April 2023 and April 2025. Of these patients, 12 were trigeminal neuralgia (46%), 6 were occipital neuralgia (23%), 4 were trigeminal neuropathic pain (15%), and 4 were PIFP (15%) patients. In our study, the landmark-based GONB technique was used to determine the greatest tenderness to palpation (TTP) area. A standard 2.5 mL mixture of 30 mg 2% lidocaine and 4 mg dexamethasone was injected bilaterally as a single dose into the nerve region of all patients. After GONB, all patients were routinely contacted by phone or addressed face to face once a week for the first month and monthly thereafter, and medical changes were recorded with a standard-case follow-up form file. The case follow-up form allowed regular monitoring of parameters, such as the Visual Analog Scale (VAS), self-assessment scales for patients’ clinical responses, sensitivity to triggers, possible side effects, duration of effect, and the number of analgesics used. **Results**: A positive response with at least 50% overall improvement compared to the patient’s baseline level was found in 22 of 26 patients. Response to treatment was observed in 10 patients in the trigeminal neuralgia group (83%), 3 patients in the trigeminal neuropathic pain (75%) and PIFP groups (75%), and all in the occipital neuralgia group (100%). There was no statistically significant difference in response rates between the diagnostic groups. A significant difference was found in terms of response rates according to gender (*p* = 0.022). Accordingly, while response was observed in all 15 female patients, response was observed in 7 of 11 male patients (64%). Pre-GONB VAS values of those responding to treatment were found to be higher. Patients with positive responses to GONB had a significantly higher median value of the VAS total score (5; 95% CI: 1.83–4.52) in comparison to those with negative responses (8.32; 95% CI: 8.17–12.12) (*p* < 0.001). Post-GONB Intensity (VAS) and Post-GONB sensitivity to triggers decreased significantly (*p* < 0.001, *p* < 0.001). In those who responded, the decrease in analgesic use after GONB compared to before was statistically significant in the first and second months (*p* < 0.001, *p* < 0.003, respectively). Although the decrease continued in the third month, this difference did not reach statistical significance (*p* = 0.551). **Conclusions**: GONB reduces the duration, frequency, and intensity of headaches, and the need for acute analgesic use in CN and PIFP patients.

## 1. Introduction

Headache disorders are among the most common neurological conditions and represent a significant global health burden [1]. Data from the Global Burden of Disease Study indicate that migraine is the second leading cause of years lived with disability (YLD) worldwide, particularly affecting individuals in their most productive years [2]. This growing burden highlights the limitations of current treatment approaches and the need for more accessible and tolerable alternatives.

In recent years, peripheral nerve blocks—especially occipital nerve blocks (ONBs)—have gained attention as a promising treatment for patients with headache disorders that are refractory to conventional therapies. These interventions are minimally invasive and may be especially useful in cases where pharmacological treatments are ineffective or poorly tolerated [3]. Clinical studies and systematic reviews have demonstrated that ONBs can result in significant reductions in headache frequency, intensity, and duration, often with a rapid onset and relatively few side effects [4,5]. ONBs are used as a treatment option for primary headaches, such as cluster headaches involving the first branch of the trigeminal nerve [6,7]. Data on its effectiveness in craniofacial neuralgias (CN) other than occipital neuralgia (ON), trigeminal neuropathic pain (TNP), and persistent idiopathic facial pain (PIFP) are limited.

Although some data are emerging regarding the mechanism of action of ONBs, the exact mechanisms remain unclear. The greater occipital nerve (GON) originates from the dorsal branches of the first three cervical roots (C1–3). It is transmitted to the spinal nucleus of the trigeminal, forming the cervical–trigeminal complex (TCC) [8,9]. This intricate functional structure comprises the Aδ- and C-type nociceptive fibers of the ophthalmic nerve, as well as the C2 root, involving relays between primary- and secondary-order neurons within the afferent pathways. The research indicates that nociceptive extracranial collateral branches of the trigeminal nerve provide innervation to the dura mater [10]. This pathway, referred to as the trigeminovascular system, is critically involved in the pathophysiology of primary headache disorders, notably migraine and cluster headaches [11]. Local perineural application of anesthetics and steroids for blockade can cause direct depolarization and inhibition of neural excitability. GON block (GONB) is thought to modulate the excitability of second-order neurons receiving input from both trigeminal and cervical afferents [12,13]. GONB affects occipital pain and other craniofacial pain by blocking pain afferents via the TCC.

The role of an ONB in treating CN has received significant attention in recent years, as evidenced by the growing literature investigating its efficacy and applications [14,15,16,17,18,19].

In facial pain syndromes targeting TCC, the topical bupivacaine application technique near the sphenopalatine foramen using an endoscopically assisted cotton ball placement of pterygopalatine ganglion block is one of the alternative intervention techniques [20]. In addition to invasive techniques, such as the infrazygomatic approach, it is accepted that the pterygopalatine ganglion blockade can be used transorally to reduce the risks associated with the procedure due to the complex structure of the surrounding neurovascular anatomy [21].

It has been shown that GONB can significantly reduce pain intensity and analgesic medication consumption in migraine [22]. Again, another study has shown that it reduces calcitonin gene-related peptide (CGRP) levels compared to the placebo [23]. GONB data on medication overuse (MOH), triptan overuse, or analgesic overuse are mostly limited to migraine-related studies. Apart from the limited data on the effect of GONB on MOH in ON patients, there is no study showing a change in analgesic use with a single application of GONB in CN [24,25,26].

Our study aims to evaluate the potential benefits of GONB in treating CN, to monitor changes in analgesic use, and to determine the efficacy of a single injection with similar doses and bilateral applications as a treatment protocol. In line with these goals, the potential benefits of GONB, a more minimally invasive treatment method, as well as the need for continued research into optimizing treatment protocols and minimizing side effects are also emphasized. In summary, we hypothesized that GONB would reduce pain and analgesic use in CN, with varying efficacy across subtypes.

## 2. Materials and Methods

This is a retrospective chart review that examined the medical records of patients who applied to Izmir University of Economics Hospital and Dr. Turan Poyraz Headache Clinic due to facial pain and cranial neuralgia between April 2023 and April 2025. Although data analysis was conducted retrospectively, the patients had been prospectively followed using a standardized-case follow-up form from the time of GONB administration. This form included predefined criteria to assess response, adverse events, analgesic use, and pain intensity at regular intervals. Therefore, this study combines a retrospective design with prospectively structured and standardized clinical data.

People who were over 18 years of age, had at least one of the craniofacial pain syndromes, had GONB performed, and had appropriate medical documentation were included in this study. Detailed inclusion criteria were as follows:

Diagnosed with trigeminal neuralgia (TN), TNP, PIFP, or ON according to ICHD-3:

- Having received a single application of GONB for acute pain exacerbations;
- Having complete follow-up data in the file for at least 3 months;
- At least one preventive medication and over 18 years old.

We excluded patients who had botulinum neurotoxin treatment in the past 3 months, multiple nerve blocks, other types of headaches, or were under 18 years old.

### 2.1. Study Procedures

To provide a classification system for painful lesions of the cranial nerves and other facial pain based on a consensus between the International Headache Society (IHS) and the International Association for the Study of Pain (IASP), chapter 13 of the International Classification of Headache Disorders, third edition (IHCD-3) is presented under the title painful lesions of the cranial nerves and other facial pain.

All patients had been seen by a headache specialist (TP) who established a diagnosis of CN, including TN (IHS 13.1.1), painful TNP (IHS 13.1.2), ON (IHS 13.4), and PIFP (IHS 13.12), according to the current ICHD-3 criteria [27].

This study’s primary outcome was a positive clinical response, defined as a 50% or greater reduction in pain intensity on the VAS compared to baseline, reported within the first week following the GONB. Secondary outcomes included changes in the number of monthly analgesic tablets, reduction in trigger sensitivity scores, duration of effect, and the presence or absence of side effects. These outcomes were tracked using the standardized-case follow-up form at each follow-up time point.

### 2.2. Methodological Limitation

The retrospective nature of the analysis may introduce selection bias, despite the use of prospectively structured data collection.

### 2.3. Ethics Approval

All participants provided written informed consent to participate in this study protocol, which was approved by the Non-Interventional Ethics Committee of Izmir Bakırçay University (Approval No. 2241, Decision No. 2253, dated 14 May 2025).

### 2.4. Greater Occipital Nerve Block

In our study, the landmark-based GONB technique was used. GONB can be localized superficially by identifying a point in the medial third of an imaginary straight line between the occipital protuberance (inion) and the mastoid prominence, or approximately 2 cm lateral and 1.5–2.0 cm below the inion. Injection sites were determined for ipsilateral pain by identifying the most incredible TTP along the superior nuchal line in the general vicinity of the target GON. The occipital artery proceeds laterally to the GON. During the injection, attention was paid to the pulsation of the artery, and the application was made medially to the artery [28,29].

### 2.5. Injection Technique

The injection technique was applied uniformly in all patients. A 2.5 mL mixture of 30 mg 2% lidocaine and 4 mg dexamethasone was injected into the nerve region, a dose similar to that in previous studies [14,29,30]. The needle was withdrawn in the same position and pressure was applied with gauze to prevent bleeding. The same procedure was used for the other side. The procedure was terminated in stable patients and they were discharged from the clinic [29,30]. Although pain localization was typically unilateral, bilateral GONB was performed in all patients to minimize the potential impact of contralateral sensitization through the TCC and to achieve more complete modulation of central pain pathways. This decision was based on the existing evidence suggesting that bilateral application may enhance efficacy, even in cases of unilaterally perceived craniofacial pain.

### 2.6. Study Design

This is a chart review based on a case follow-up form and no experimental design planning was performed. The case follow-up form is a documentation created for headaches and is quite strictly standardized. Bilateral block application was performed for patients with CN. The first dose of the lidocaine and dexamethasone mixture was applied with the same dose and content for patients over the age of 18 years. Therefore, confusion regarding the application technique, the content of the medication applied, and unilateral/bilateral side applications was eliminated. By including cases with a single GONB application in the study design, design problems such as insufficient variables recorded in the graph, the presence of excessive or confusing comorbidities, and/or the presence of confusing factors that will sufficiently reduce the validity of the data from the graph were prevented [31]. After GONB, all patients were routinely contacted by phone or in person on days 0, 3, 7, 14, 21, and 30, and monthly thereafter, and any medical changes were recorded using a standard-case follow-up form. The case follow-up form allows regular monitoring of parameters, such as the Visual Analog Scale (VAS) and susceptibility to triggers, which are self-assessment scales of patients’ clinical responses, as well as possible side effects, duration of effect, and monthly monitoring of the number of analgesics used. Since this study is a retrospective cohort study based on prospective follow-up, including patients with complete study data, there are no dropout rates and missing follow-up data.

### 2.7. Statistical Methods

Categorical measurements were summarized as numbers and percentages, and numerical measurements were summarized as mean and standard deviation (median and minimum–maximum where necessary). In cases where there was no expected value problem in comparison of categorical measurements between groups, the Pearson Chi Squared test was used. In cases of an expected value problem, Fisher’s Exact test was used. The Shapiro–Wilk test and normality graphs (Q-Q plots) were used to examine whether the numerical measurements followed a normal distribution. To compare numerical measurements according to ONB response, an independent-groups *t*-test was used in case assumptions were met, and a Mann–Whitney U test was used in case assumptions were not met. A dependent-groups t-test was used to compare VAS measurements before and after ONBs. In general, when comparing numerical measurements according to diagnosis, a one-way analysis of variance was used if the case assumptions were met, and the Kruskal–Wallis test was used if the case assumptions were not met. The Tukey test, Games–Howell test, or Bonferroni corrected Mann–Whitney U test was used for multiple comparisons. The SPSS 20.0 package program was used for the statistical analysis of the data. A statistical significance level of 0.05 was used in all tests.

The power analysis of this study was carried out using G*Power 3.1.9.2 (Universität Kiel, Kiel, Germany) [32]. Since this study involved four diagnostic subgroups, the F test was selected as the test type, and “ANOVA: fixed-effects, omnibus, one-way” was chosen as the statistical method. Based on preliminary data from a pilot group, the expected common standard deviation was estimated at 10 units on the VAS, and the minimum detectable mean difference between groups was set at 12 units, yielding an effect size of f = 0.521. Using a power (1–β) of 0.95 and an alpha level of 0.05, the minimum required sample size was calculated to be 24 patients. Considering potential dropout or missing data, the final target sample size was increased by 10%, resulting in a total sample of 26 patients, which was achieved. This power analysis supports the adequacy of the sample size for detecting differences across diagnostic groups with moderate to large effect sizes. Cohen’s d and Eta Squared effect size calculation methods used in the study are presented in Table S1 in the Supplementary Materials section, and Cramer’s V effect size calculation method is presented in Table S2.

## 3. Results

This study included 26 patients with CN who presented with an acute pain attack and were provided a single, bilateral GONB at the same dose as standard. Of these patients, 12 were TN (46%), 6 were ON (23%), 4 were TNP (15%), and 4 were PIFP (15%). The clinical characteristics of the patients are presented in Table 1.

The mean age of the patients was 58.9 ± 13.0 years (range: 26–76 years); 15 of them were female (58%) and 11 were male (42%). Nineteen patients (73%) were married, and seven (27%) were single. The sociodemographic and diagnostic characteristics of the patients are presented in Table 2. Since concomitant medications were used according to diagnosis (e.g., gabapentinoids and antidepressants were used in almost all patients diagnosed with TN or FP, while antidepressants were mainly used in patients diagnosed with TNP or ON), there is no patient profile in this study to investigate the effect of medication use on the outcome.

### 3.1. Response Rates

**Figure 1:**
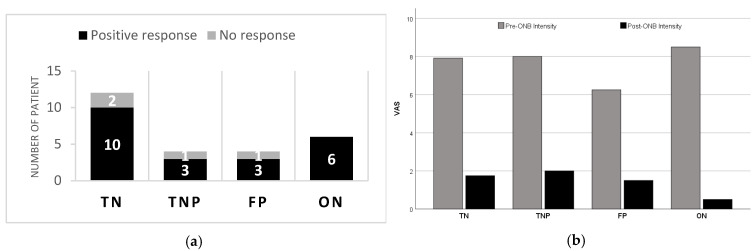
The percentage of patients in each diagnostic group reported ≥50% clinical improvement after GONB. (**a**) The rate of patients responding to pain over 50% according to diagnosis groups. The height of bars indicates total number of patients; the black parts indicate patients with success. (**b**) Comparison of mean VAS scores before and after GONB according to diagnosis groups. TN: trigeminal neuralgia, TNP: trigeminal neuropathic pain, FP: persistent idiopathic facial pain, ON: occipital neuralgia, ONB: occipital nerve block, and VAS: Visual Analog Scale.

A positive response with at least 50% overall improvement compared to the patient’s baseline level was found in 22 of 26 patients (85% [95% CI: 71–98%]; Figure 1). Response to treatment was observed in 10 patients (83% [95% CI: 62–100%]) in the TN group, 3 patients in the TNP (75% [95% CI: 33–100%]) and FP (75% [95% CI: 33–100%]) groups, and all in the ON group (100% [95% CI: 100–100%]). No statistically significant difference was found in terms of response rates between the diagnostic groups (*p* = 0.623, Cramer’s V effect size = 0.16). The lack of statistical difference between diagnostic groups may be due to the relatively small sample size, limiting the generalizability of subgroup comparisons. After assessing all patients, a significant difference was found in response rates according to gender (*p* = 0.022, Cramer’s V effect size = 0.5). Accordingly, while a response was observed in all 15 female patients, a response was observed in 7 of 11 male patients (64%). Data affecting response rates are presented in Table 3.

**Figure 2:**
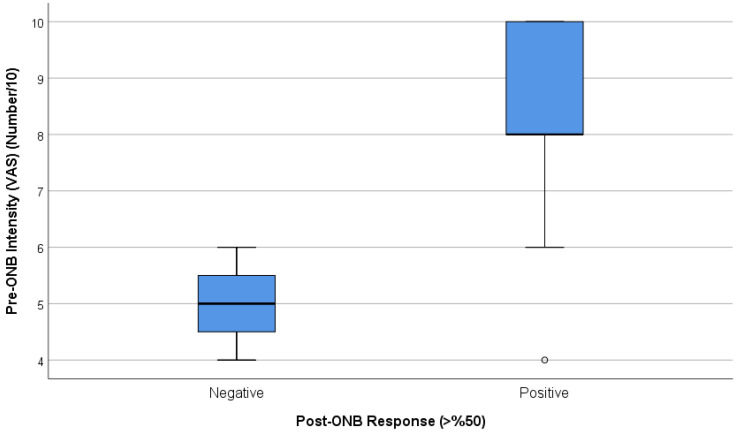
Comparison of pre-GONB VAS scores between patients with and without clinical responses. Patients who reported a ≥50% reduction in pain (responders) had significantly higher baseline pain scores than non-responders. Data are presented as median and confidence interval values. VAS: Visual Analog Scale.

Pre-GONB VAS values of those responding to treatment were found to be higher. Patients with positive responses to GONBs had a significantly higher median value of VAS total score (8.32; [95% CI: 8.17–12.12%]) in comparison to those with negative responses (5; [95% CI: 1.83–4.52%]) (*p* < 0.001, Cohen’s d effect size = 2.59) (Figure 2).

Analgesic use before GONB is not statistically significant in relation to factors such as tenderness over the GON and susceptibility to triggers (Table 4).

### 3.2. Clinical Improvement

**Figure 3:**
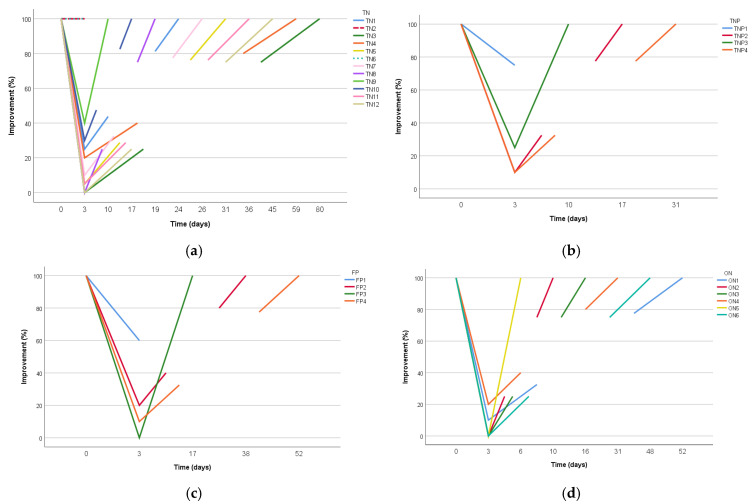
Individual response timelines to GONB in each diagnostic subgroup. Time graph of the response elicited by a GONB. (**a**) Trigeminal neuralgia (TN) group, (**b**) trigeminal neuropathic pain (TNP) group, (**c**) persistent idiopathic facial pain (PIFP) group, and (**d**) occipital neuralgia (ON) group. Each line represents the duration of a patient’s reported clinical improvement (in days). Variation in response patterns across diagnostic groups reflects heterogeneity in underlying pathophysiology and treatment responses.

Clinical improvement was monitored by at least a 50% improvement, as reported verbally by telephone or face-to-face interviews. The mean duration of clinical improvement (only considering patients who showed at least 50% improvement on day 3) was 22.3 ± 19.5 days for all patients. The median duration was 26.3 ± 18.5 days (range: 3–77 days) for patients who only improved (*n* = 22). The difference in the mean for all patients was that the duration value was taken as 0 for those who did not improve. There was no statistically significant difference in the mean duration of clinical improvement between diagnostic groups, both in all patients and in responding patients (Table 5). The maximum follow-up period for all patients was determined as 3 months, and individual responses to GONB in patients constituting diagnostic groups (Figure 3).

### 3.3. Comparison of Diagnostic Groups

A statistical difference was found between the mean ages of the diagnostic groups. Accordingly, the mean age of TNP patients was lower than that of the other groups. In addition, the mean age of the TN group was higher than that of the ON group. (The reason why the mean age of the FP group was not found to be statistically higher than the ON group is that there were fewer patients in the FP group). Concomitant disease is not observed in the TN and FP groups, but is present in more than 50% of the TNP and ON groups. Data related to the comparison of the sociodemographic and clinical characteristics of the patients included in this study with their diagnostic groups are presented in Table 6.

VAS values showed an average decrease of 72% in TN patients, 70% in TNP patients, 73% in FP patients, and 94% in ON patients after ONB compared to before ONB. Although a higher numerical change was seen in the ON group, this difference could not be observed statistically (*p* = 0.552, Eta-squared effect size = 0.09). When the VAS values of the diagnostic groups before and after GONB were compared, no statistical difference was found between the VAS values of the groups at both times (for before GONB, *p* = 0.346, Eta-squared effect size = 0.14, and for after GONB, *p* = 0.507, Eta-squared effect size = 0.10). On the other hand, VAS values decreased statistically significantly after GONB in all groups compared to before GONB (Table 7).

**Figure 4:**
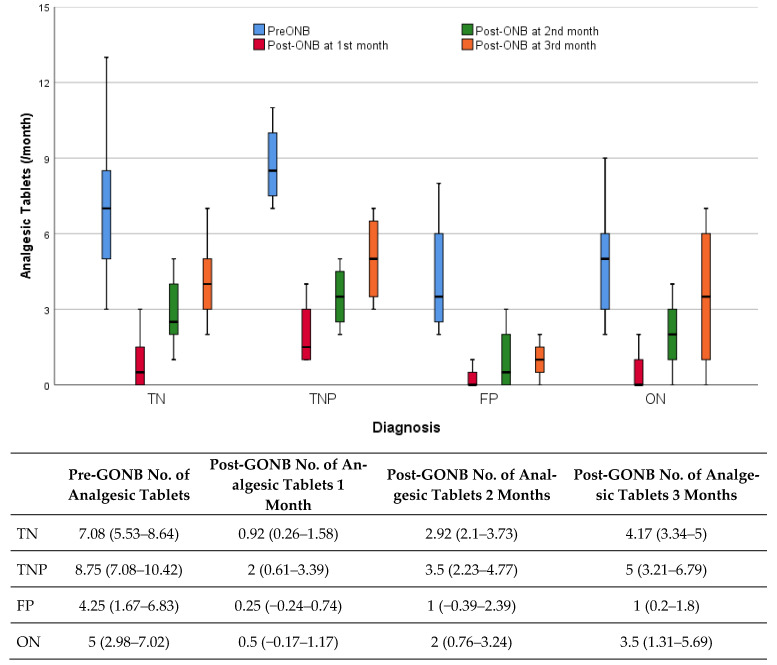
The monthly number of analgesic tablets used across groups before and after GONB. Box plot includes mean and 95% confidence intervals. A box plot graph illustrating the distribution of monthly analgesic tablet quantities, categorized by month and group. The bar heights show the median number of tablets, along with the confidence interval.

It would be correct to look at the agreement rather than the difference between the measurements after the presence of response and GONB. In those who received a response, the decrease in the percentage of pain after GONB compared to before, Post-GONB Intensity (VAS), and Post-GONB Susceptibility to triggers decreased statistically significantly (*p* < 0.001, *p* < 0.001, *p* < 0.001, respectively) (Table 8). In those who responded, the decrease in analgesic use after GONB compared to before was statistically significant in the 1st and 2nd months (*p* < 0.001, *p* < 0.003, respectively). Although the decrease continued in the 3rd month, this difference did not reach statistical significance (*p* = 0.551) (Table 8, Figure 4).

### 3.4. Safety and Side Effects

The injection procedure was rated as painful in only two of the patients. One of these patients was in 12 TN patients (8%) and the other was in 4 FP patients (25%). Mild and transient side effects associated with GONB were observed in 3 of the 24 patients. Of the patients experiencing side effects, 2 (17%) were in the TN group and 1 (25%) was in the FP group. Cranial burning or heat sensation was observed in all three patients. All side effects entirely resolved within 3 days at most. Local bleeding after syringe removal accompanied this side effect in 1 patient, Heart Rate Variability in 1 patient, and both Heart Rate Variability and Tenderness over the injection site in 1 patient.

## 4. Discussion

The present study used GONB as a single-dose acute attack intervention for CN. We showed that GONB for multimodal pain treatment may provide additional pain relief in patients with CN, since some of the patients had received conventional treatments for their pain with a neuropathic component for at least 3 months and reported dissatisfaction. Various therapies, including gabaphentinoids, antiepileptics, and antidepressants, modulate neuropathic pain by affecting the trigeminal nucleus [33]. It was found that bilateral GONB reduced both the intensity and duration of headaches, the number of headache days, and the need for acute attack treatments. Patients treated with GONB experienced statistically significant reductions in headache intensity, duration, number of headache days, VAS scores, sensitivity to triggers, and the number of analgesic uses compared to their pretreatment levels. There were no differences between the CN groups in terms of variables such as decreased VAS scores, reduced sensitivity to triggers, decreased tenderness over the GON, and decreased analgesic use with GONB. GONB did not show any significant side effects in all groups. All observed mild side effects were transient.

In our study, the response of female CN patients to GONB was more pronounced. All female patients (100%) reported a positive response, compared to 64% of male patients, and this difference was statistically significant. Several factors may contribute to this observation. Previous studies have suggested that gender differences in pain perception and modulation affect pain thresholds and treatment response [34,35]. Although the current study was not designed to explore gender-specific mechanisms, these findings highlight the need for further investigation into sex-related differences in response to interventional headache treatments, such as GONB.

The findings of this study include a decrease in VAS scores to 50% of the baseline values (*p* = 0.0001) in CN patients undergoing GONB. Secondary findings confirm our hypothesis that analgesic consumption was reduced.

In another open-label retrospective study conducted for CN, it was observed that certain methodological variables were present, including multiple GON applications to non-responders, bilateral and unilateral applications to some patients, and variable content of local anesthetic and steroid doses in the applied medication [14]. In our study, we controlled for such confounding variables by using the same standardized methodology across all patient groups. A single application was applied bilaterally to all patients, regardless of their response. Anesthetic and steroid contents, doses, and application amounts were the same. When considering differences across diagnostic groups, our results show the highest response rates in patients with ON, those with TN, TNP, and PIFP. The greater responsiveness in ON and TN may reflect more peripheral, segmentally organized pain generators directly influenced by GONB. In contrast, the lower response observed in PIFP may be due to its more central sensitization profile, with less direct involvement of GON-mediated pathways. While all groups demonstrated a statistically significant reduction in VAS scores post-GONB, the duration and magnitude of clinical benefit appeared to vary, suggesting that different craniofacial pain syndromes may require tailored interventions. These group-specific differences should be further explored in future prospective studies.

In a study in which a single GONB application was performed in patients with primary headaches, with a mixture of 3 mL of 2% lidocaine and 80 mg of methylprednisolone, it was shown that the pain response continued for an average of 30 days for migraine patients and an average of 21 days for cluster headache patients [30]. Our study observed that this period lasted between 16 (FP) and 32 (TN) days, with no significant difference between the groups. However, a limitation of this study is the lack of structured longitudinal tracking of attack frequency beyond the initial response window. Although analgesic consumption and VAS scores were monitored monthly for three months, attack frequency was not systematically documented using patient diaries or validated headache calendars. Therefore, sustained treatment efficacy over more extended periods remains uncertain. Future prospective studies should incorporate standardized headache diaries to accurately assess long-term changes in attack frequency and recurrence patterns.

There are no studies on an effective local anesthetic dose for GONB. However, studies generally suggest the use of 1–2% lidocaine (10–20 mg/mL) and/or 0.25–0.5% bupivacaine (2.5–5 mg/mL) for effective GONB [36,37]. Our study data show that lidocaine is an effective local anesthetic for CN, as it is for other primary headaches [14,15,30,38,39]. Some studies suggest a slower onset of action and a longer duration of action with bupivacaine [24]. In our study, we preferred patient groups with similar local anesthetic use because the drug was more readily available, and to compare the data from the study by Jürgens et al. with our data [14]. Similarly, standard doses of dexamethasone were preferred as a corticosteroid. However, we cannot exclude the fact that long-acting corticosteroids, such as triamcinolone, may be more advantageous.

We added corticosteroids to local anesthetics because of their effects, such as reducing inflammation by inhibiting the synthesis or release of several proinflammatory substances, some cytokines, and other acute phase reagents following trigeminal activation, direct membrane stabilizing effect, reversible inhibition of nociceptive C-fiber transmission, and modulation of nociceptive input within dorsal horn substantia gelatinosa neurons [22,35,40].

As a result, in our study, a mixture of 30 mg 2% lidocaine and 4 mg dexamethasone in 2.5 mL was injected into both GONs. Injection sites were determined for ipsilateral pain by identifying the TTP along the superior nuchal line in the general vicinity of the target GON. Here, the GON injection technique of Afridi et al. was combined with the injection technique of Tobin and Flitmann [24,30]. In our study, a landmark-based GONB was applied bilaterally, although TTP was not bilateral. In general, there is insufficient evidence regarding the superiority of unilateral or bilateral GONB over the other. However, it is thought that a bilateral approach may provide more effective intervention in central sensitivity due to the bilateral relationship of the occipital nerves within the TCC, which exhibit atypical distribution [41,42].

GON tenderness or a positive Tinel sign over the GON was significantly associated with a positive response to injection. Since this may be useful as a rapid assessment test for which patients’ GON blockade will be effective, this clinical assessment was also performed in our study [14,24]. It was observed that the response was greater in patients with a positive Tinel sign on the ipsilateral side to the pain. This difference did not reach statistical significance, which may be attributed to our study’s relatively small sample size.

An increasing number of studies are being conducted on the effectiveness of different approaches for GONB. These studies mostly compare ultrasound-guided, landmark-based, and radiofrequency-combined GONB treatments in patients with chronic migraine [25,41,42,43].

A retrospective cohort study evaluating the efficacy of ultrasound (US)-guided continuous radiofrequency ablation (CRFA) of the proximal GON in 18 patients with refractory ON found that CRFA effectively reduced attack frequency and pain in refractory ON [44].

There are limited data on the effect of GONB on analgesic overuse or MOH in CN. Studies primarily include data on the efficacy of GONB in the presence of MOH in ON [24,25]. The increase in the failure rate associated with MOH was greater in migraine patients than in those with ON. It was statistically significant and high, especially in treatment-resistant migraine patients [24]. One of the few studies investigating the efficacy of GONB on CN, a study conducted by Jürgens et al., although similar to our study in terms of patient grouping, did not report an assessment of the efficacy of GONB on MOH due to fundamental methodological differences [14]. Methodologically, differences such as the variable application frequency of GONB, unilateral application to some patients, different drug doses, and the adoption of the method applied by Afridi et al. as the application technique may have caused a change in the study results [30]. Afridi et al. showed that GONB is more effective in migraine patients with more persistent MOH [30]. GONB with lidocaine was also found to be effective in the treatment of triptan-overuse headaches [26]. However, repeated applications were found to be more effective than a single application. Our study showed that monthly symptomatic analgesic use was reduced for up to 3 months with a single injection. This reduction was shown to be statistically significant during the first 2 months. The difference between the studies may be related to the difference in disease groups. It can be said that GONB is an effective method especially in MOH observed in CN and refractory migraine patients. We believe that our study will provide valuable insights into the literature by evaluating the effectiveness of GONB on CN and its impact on MOH. Jürgens et al. reported that GONB is less effective in FP than in CN. The researchers attributed the low effect of GONB to the lack of a consistent structure in the PIFP, the unchanged cortical somatosensory representation of the face, and the absence of the impact on quantitative sensory testing modalities [14]. Several studies have shown no evidence of a pathophysiological role of neurovascular compression of the trigeminal dorsal root entry zone in PIFP, or, as it was previously termed, atypical facial pain (AFP). The relatively low positive response observed in PIFP patients may be due to variables such as central sensitivity, heterogeneity of pathophysiology, or placebo response [45,46].

A study in which QST (Quantitative Sensory Test) application to PIFP patients showed sensory abnormalities in small fibers in 55% of the patients highlighted changes in the nociceptive system at the central level [47]. Recent studies have reported that PIFP is a general dysfunction of a series of nociceptive systems, ranging from somatosensory dysfunction to changes in intracortical modulation, and may lead to alterations in dopamine systems related to pain transmission and modulation, accompanied by increased neuronal excitability at the brainstem level [48,49,50]. In a retrospective study by Can and Akkaya, a US-guided suprazygomatic block was found to have a successful analgesic effect on refractory PIFP patients [51]. In our research, GONB was an effective and safe method in FP, as in other FP syndromes. This also suggests the existence of a complex interaction between FP and TCC. This effect may be due to the placebo response observed in invasive drug efficacy studies. However, the long analgesic-effect duration observed along with the fact that FP provides a successful analgesic effect on other blockade methods, such as suprazygomatic block, may facilitate an objective response evaluation.

Side effects were rarely observed in our study. These side effects were mild, transient, and completely reversible. Studies on larger study groups report more serious side effects due to errors that can occur, such as the intravenous injection of local anesthetics [14,24,52]. However, landmark-based GONB, made by experienced hands, is practical when combined with TTP.

### 4.1. Limitations

This study has several limitations:

- The sample size was relatively small, limiting the generalizability of subgroup comparisons.
- The retrospective nature of the analysis may introduce selection bias, despite the use of prospectively structured data collection.
- The absence of structured headache diaries limited the accurate assessment of long-term attack frequency.
- The placebo effect cannot be excluded, although the observed improvements in some patients lasted several weeks, exceeding typical placebo durations.

### 4.2. Future Directions

Future research should focus on conducting randomized, double-blind, placebo-controlled trials to validate the efficacy of GONB across different craniofacial pain syndromes. Incorporating larger, more diverse patient populations and using structured headache diaries or electronic pain tracking tools will help capture long-term outcomes more accurately. Additionally, comparative studies examining the effects of unilateral versus bilateral GONB and different local anesthetics and steroid types or dosages may offer insights into optimizing block protocols. Exploring predictive biomarkers of response, such as GON tenderness or CGRP levels, could also support personalized intervention strategies.

## 5. Conclusions

This study shows that GONB is promising in TN and ON, but requires further study for TNP and PIFP. GONB reduced pain and analgesic use in TN and ON, but efficacy in TNP and PIFP remains uncertain due to factors such as small sample sizes. Due to the rare and mild side effects and long duration of effect, we recommend that GONB, a minimally invasive method, be applied before invasive approaches in this patient group. Further biomarker-based studies comparing GONB in larger and more diverse headache cohorts, as well as in randomized, double-blind, placebo/sham-controlled trials, are needed to confirm these findings. These results support the inclusion of GONB as part of an individualized treatment plan for patients with craniofacial neuralgias, particularly when medication overuse or trigger sensitivity complicates management.

## Figures and Tables

**Table 1:** Clinical characteristics of the included patients.

ID	Age (Years)	Gender	Diagnosis	Branch	Side	Duration (Years)	Current Medication
TN1	72	F	TN	V2, V3	Right	15	GABA phentinoids
TN2	65	M	TN	V1, V2, V3	Right	8	GABA phentinoids
TN3	68	F	TN	V2, V3	Right	4	GABA phentinoids + Antidepressants
TN4	62	F	TN	V2	Left	1	GABA phentinoids + Antidepressants
TN5	60	F	TN	V2, V3	Right	6	GABA phentinoids + Antidepressants
TN6	66	M	TN	V2	Right	5	Antiepileptics
TN7	58	F	TN	V2	Left	2	GABA phentinoids + Antidepressants
TN8	74	M	TN	V3	Right	11	Antiepileptics + Antidepressants
TN9	62	F	TN	V3	Right	2	GABA phentinoids + Antidepressants
TN10	67	F	TN	V2, V3	Right	4	GABA phentinoids
TN11	65	F	TN	V3	Left	5	GABA phentinoids + Antidepressants
TN12	67	F	TN	V2, V3	Right	10	GABA phentinoids + Antidepressants
TNP1	52	M	TNP	V2	Right	1	Antiepileptics + Antidepressants
TNP2	48	M	TNP	V2	Right	3	Antiepileptics + Antidepressants
TNP3	26	M	TNP	V3	Right	1	Antiepileptics + Antidepressants
TNP4	37	F	TNP	V3	Left	4	GABA phentinoids + Antidepressants
FP1	76	M	PIFP		Right	8	Antiepileptics
FP2	68	F	PIFP		Both	12	GABA phentinoids + Antidepressants
FP3	62	F	PIFP		Both	5	GABA phentinoids + Antidepressants
FP4	67	F	PIFP		Left	3	GABA phentinoids + Antidepressants
ON1	33	F	ON		Left	2	None
ON2	74	M	ON		Right	3	Antidepressants
ON3	52	M	ON		Right	7	Antidepressants
ON4	48	M	ON		Left	1	None
ON5	44	F	ON		Left	1	None
ON6	59	M	ON		Right	3	None

Abbreviations: TN (trigeminal neuralgia), TNP (trigeminal neuropathic pain), FP (facial pain), ON (occipital neuralgia), F (female), and M (male).

**Table 2:** Sociodemographical and diagnostic characteristics of the included patients.

Characteristics	Mean ± SD	Median (Min–Max)	*n*%
Age	58.9 ± 13.0	62 (26–76)	
Gender Female Male			15 (58%) 11 (42%)
Diagnosis TN TNP FP ON			12 (46%) 4 (15%) 4 (15%) 6 (23%)
Marital status Married Single			19 (73%) 7 (27%)
Side of pain Right Left Bilateral			16 (61%) 8 (31%) 2 (8%)
Disease duration (year)	4.9 ± 3.8	4 (1–15)	

Abbreviations: TN (trigeminal neuralgia), TNP (trigeminal neuropathic pain), FP (facial pain), ON (occipital neuralgia), and SD (standard deviation).

**Table 3:** Sociodemographical and diagnostical characteristics of included patients and their associations with response rates.

	Positive Response	No Response	*p*	Effect Size
Age, Mean ± SD	57.9 ± 13.4	64.7 ± 9.8	0.340	0.59 *
Gender*, n* (%) ** **Female ** **Male	15 (100%) 7 (64%)	0 (0%) 4 (36%)	0.022	0.50 **
Diagnosis*, n* (%) ** **TN ** **TNP ** **FP ** **ON	10 (83%) 3 (75%) 3 (75%) 6 (100%)	2 (17%) 1 (25%) 1 (25%) 0 (0%)	0.623	0.16 **
Marital status*, n* (%) ** **Married ** **Single	15 (79%) 7 (100%)	4 (21%) 0 (0%)	0.546	0.26 **
Side of pain*, n* (%) ** **Right ** **Left ** **Bilateral	12 (75%) 8 (100%) 2 (100%)	4 (25%) 0 (0%) 0 (0%)	0.347	0.24 **
Disease duration (year), Mean ± SD	4.8 ± 3.9	5.5 ± 3.3	0.732	0.20 *
Concomitant disease*, n* (%) ** **Present ** **Absent	16 (80%) 6 (100%)	4 (20%) 0 (0%)	0.542	0.23 **
Current medication*, n* (%) ** **GABA phentinoids ** **Antiepileptics ** **Antidepressants ** **None ** **GABA phentinoids + Antidepressants ** **Antiepileptics + Antidepressants	2 (67%) 0 (0%) 2 (100%) 4 (100%) 11 (100%) 3 (75%)	1 (33%) 2 (100%) 0 (0%) 0 (0%) 0 (0%) 1 (25%)	0.014	0.34 **

Abbreviations: TN (trigeminal neuralgia), TNP (trigeminal neuropathic pain), FP (facial pain), ON (occipital neuralgia), SD (standard deviation), * Cohen’s d was calculated for effect size, and ** Cramer’s V was calculated for effect size.

**Table 4:** Factors affecting the differences between pre-GONB response groups.

	Response Positive	No Response	*p*	Effect Size
Pre-GONB No. of Analgesic Tablets Mean ± SD	6.6 ± 3.0	5.5 ± 1.7	0.492	0.44 *
Pre-GONB Tenderness over GON*, n* (%) ** **Ipsilateral ** **Ipsilateral and Contralateral ** **N.A.	20 (91%) 1 (5%) 1 (5%)	2 (50%) 2 (50%) 0 (0%)	0.099	0.33 **
Pre-GONB Intensity (VAS) (Number/10) Mean ± SD	8.3 ± 1.6	5.0 ± 0.8	0.001	2.59 *
Pre-GONB Susceptibility to Triggers (Number/10) Mean ± SD	6.0 ± 3.5	7.0 ± 1.8	0.584	0.36 *

Abbreviations: GONB (greater occipital nerve block), N.A. (not applicable), SD (standard deviation), VAS (Visual Analog Scale), * Cohen’s d was calculated for effect size, and ** Cramer’s V was calculated for effect size.

**Table 5:** Comparison of clinical improvements of the included patients with the diagnostic groups.

	Diagnosis					*p*
	TN	TNP	FP	ON	Total	
n (all patients)	12	4	4	6	26	
Post-GONB duration of improvement (days)	26.4 ± 23	12.2 ± 12	24.5 ± 21.8	19.2 ± 15.4	22.3 ± 19.5	0.668
n (patients with a positive response)	10	3	3	6	22	
Post-GONB duration of improvement (days)	31.7 ± 21.4	16.3 ± 10.7	32.7 ± 17.6	19.2 ± 15.5	26.3 ± 18.5	0.42

Abbreviations: TN (trigeminal neuralgia), TNP (trigeminal neuropathic pain), FP (facial pain), ON (occipital neuralgia), and GONB (greater occipital nerve block).

**Table 6:** Comparison of sociodemographic and clinical characteristics of included patients with diagnostic groups.

Characteristics	Diagnosis				*p*	Effect Size
	TN	TNP	FP	ON		
Age, Mean ± SD	65.5 ± 4.6	40.8 ± 11.7	68.2 ± 5.8	51.7 ± 14	<0.001	0.59 *
Gender*, n* (%) Female Male	9 (75%) 3 (25%)	1 (25%) 3 (75%)	3 (75%) 1 (25%)	2 (33%) 4 (67%)	0.166	0.25 **
Marital status*, n* (%) Marriage Single	10 (83%) 2 (17%)	2 (50%) 2 (50%)	3 (75%) 1 (25%)	4 (67%) 2 (33%)	0.661	0.17 **
Side of pain, *n* (%) Right Left Bilateral	9 (75%) 3 (25%) 0 (0%)	3 (75%) 1 (25%) 0 (0%)	1 (25%) 1 (25%) 2 (50%)	3 (50%) 3 (50%) 0 (0%)	0.131	0.23 **
Disease duration (*years),* Mean ± SD	6.1 ± 4.2	2.2 ± 1.5	7 ± 3.9	2.8 ± 2.2	0.055	0.25 *
Concomitant disease*, n* (%) Present Absent	12 (100%) 0 (0%)	1 (25%) 3 (75%)	4 (100%) 0 (0%)	3 (50%) 3 (50%)	0.003	0.39 **
Current medication*, n* (%) GABA phentinoids Antiepileptics Antidepressants (AD) None GABA phentinoids + AD Antiepileptics + AD	3 (25%) 1 (8%) 0 (0%) 0 (0%) 7 (58%) 1 (8%)	0 (0%) 0 (0%) 0 (0%) 0 (0%) 1 (25%) 3 (75%)	0 (0%) 1 (25%) 0 (0%) 0 (0%) 3 (75%) 0 (0%)	0 (0%) 0 (0%) 2 (33%) 4 (67%) 0 (0%) 0 (0%)	<0.001	0.26 **

Abbreviations: TN (trigeminal neuralgia), TNP (trigeminal neuropathic pain), FP (facial pain), ON (occipital neuralgia), SD (standard deviation), * Eta-squared was calculated for effect size, and ** Cramer’s V was calculated for effect size.

**Table 7:** Comparison of mean VAS values pre- and post-GONB with the diagnostic groups.

	TN	TNP	FP	ON	*p*	Effect Size
VAS change percentage	72.4 ± 36.6	69.8 ± 36.8	72.8 ± 37.7	94.2 ± 9.5	0.552	0.09 *
Pre-GONB VAS	7.9 ± 2.1	8.0 ± 1.6	6.3 ± 2.2	8.5 ± 1.5	0.346	0.14 *
Post-GONB VAS	1.8 ± 2.0	2.0 ± 2.2	1.5 ± 1.9	0.5 ± 0.8	0.507	0.10 *
*p*	<0.001	<0.001	<0.001	<0.001		
Effect size	3.06 **	3.13 **	2.29 **	6.53 **		

Abbreviations: TN (trigeminal neuralgia), TNP (trigeminal neuropathic pain), FP (facial pain), ON (occipital neuralgia), GONB (greater occipital nerve block), VAS (Visual Analog Scale), * Eta-squared was calculated for effect size, and ** Cohen’s d was calculated for effect size.

**Table 8:** Clinical parameters affecting the differences between post-GONB response groups.

Variables (Mean ± SD)	Positive Response	No Response	*p*
Post-GONB % of Pre-GONB Pain	10.9 ± 11.7	83.8 ± 19.7	<0.001
Post-GONB No. Of Analgesic Tablets 1 Month	0.5 ± 0.7	2.8 ± 1.3	<0.001
Post-GONB No. Of Analgesic Tablets 2 Months	2.3 ± 1.6	3.8 ± 0.5	0.003
Post-GONB No. Of Analgesic Tablets 3 Months	3.5 ± 2.2	4.2 ± 1.7	0.551
Post-GONB Intensity (VAS) (Number/10)	0.9 ± 1.1	4.8 ± 0.5	<0.001
Post-GONB Susceptibility to Triggers (Number/10)	0.5 ± 0.7	4.2 ± 1.3	<0.001
Post-GONB Painful*, n* (%) ** **Positive ** **Negative	1 (5%) 21 (95%)	1 (25%) 3 (75%)	0.289
Post-GONB Side Effects*, n* (%) ** **Positive ** **Negative	1 (5%) 21 (95%)	2 (50%) 2 (50%)	0.052

Abbreviations: GONB (greater occipital nerve block); VAS (Visual Analog Scale).

## Data Availability

No datasets were generated or analyzed during the current study.

## Supplementary Materials

The following supporting information can be downloaded at: https://www.mdpi.com/article/10.3390/jcm14145034/s1, Table S1: Effect size calculation method for Cohen’s d and Eta-squared; Table S2: Effect size calculation method for Cramer’s V.

## Abbreviations

The following abbreviations are used in this manuscript:

CN	Craniofacial neuralgia
CGRP	Calcitonin gene-related peptide
FP	Facial pain
GON	Greater occipital nerve
GONB	Greater occipital nerve block
ICHD-3	International classification of headache disorders—third edition
IHS	International headache society
MOH	Medication-overuse headache
ON	Occipital neuralgia
ONB	Occipital nerve block
PIFP	Persistent idiopathic facial pain
TCC	Trigeminocervical complex
TN	Trigeminal neuralgia
TNP	Trigeminal neuropathic pain
TTP	Tenderness to palpation
VAS	Visual analog scale
