# Supplementary material for: Efficacy of Greater Occipital Nerve Blockade in Craniofacial Neuralgia and Facial Pain Syndromes: A Retrospective Chart Review with Prospectively Collected Follow-Up Data
Source: J Clin Med. 2025 Jul 16;14(14):5034. doi: 10.3390/jcm14145034 (PMC12295901; doi:10.3390/jcm14145034)
Supplement: Supplementary file 1 [file jcm-14-05034-s001.zip › jcm-3748741-supplementary.pdf]

Table S1: Effect size calculation method for Cohen’s d and Eta squared;

Effect size calculation method	Lower limit for small effect size	Lower limit for medium effect size	Lower limit for large effect size
Cohen’s d	0.20	0.50	0.80
Eta squared	0.01	0.06	0.14

Table S2: Effect size calculation method for Cramer’s V

Limits for Cramer's V			
**Degree of freedom**	**Small effect size**	**Medium effect size**	**Large effect size**
1	0.10	0.30	0.50
2	0.07	0.21	0.35
3	0.06	0.17	0.29
4	0.05	0.15	0.25
5	0.04	0.13	0.22
